# Dickkopf 3—A New Indicator for the Deterioration of Allograft Function After Kidney Transplantation

**DOI:** 10.3389/fmed.2022.885018

**Published:** 2022-05-11

**Authors:** Antonia Schuster, Louisa Steines, Karolina Müller, Florian Zeman, Peter Findeisen, Bernhard Banas, Tobias Bergler

**Affiliations:** ^1^Department of Nephrology, University Hospital Regensburg, Regensburg, Germany; ^2^Center for Clinical Studies, University Hospital Regensburg, Regensburg, Germany; ^3^MVZ Laboratory Dr. Limbach, Heidelberg, Germany

**Keywords:** kidney transplantation, allograft survival, Dickkopf (DKK), albuminuria, glomerular filtration rate

## Abstract

Evidence of tubular atrophy and interstitial fibrosis is prognostically unfavorable and associated with a premature graft loss after kidney transplantation. Recently, Dickkopf 3 (DKK3), a profibrotic glycoprotein released by stressed tubular epithelial cells, has been identified to cause IF/TA by regulating the Wnt/β-catenin signaling and seems to engage a T-cell response. The aim of our study was to determine if a correlation between DKK3 and graft function exists and if DKK3 could be a new indicator to identify patients at risk for a deterioration in graft function. Patients, transplanted between 2016 and 2018, were analyzed with regard to DKK3 in the urine and graft function (creatinine, eGFR, albuminuria). Multivariable analyzes were used including known factors influencing graft function (PRA, donor age) to stress robustness of DKK3. The 3 and 12 month DKK3 values were significant predictors for subsequent graft function up to 36 months. An increase of DKK3 from month 3 to 12 of ≥ 25% showed a higher risk of an impaired graft function, with, e.g., a reduction in eGFR of about 9–10 ml/min in contrast to patients without intensified DKK3 increase. Induction therapy has an influence on DKK3 as patients induced with a T-cell depleting therapy showed a trend toward lower DKK3 values. In summary, our study is the first investigation of DKK3 in kidney transplant recipients and was able to show that DKK3 could forecast graft function. It is recommended to investigate the potential of DKK3 as a predictor of kidney function after transplantation in further studies.

## Introduction

Kidney transplantation remains the preferred treatment for patients with end stage kidney disease due to a better patient survival compared to dialysis. Despite good short-term results, ensuring a long-lasting graft function is an unsolved problem. To date, with serum creatinine, eGFR and albuminuria, only a few parameters are available in everyday clinical practice to monitor graft function. Even in the KDIGO guidelines, these parameters are named as the main monitoring tool (1). However, these parameters have not yet succeeded in ensuring graft survival. In literature, different biomarkers are being discussed in order to ensure a better risk assessment.

Park et al. showed that an eGFR decline of >–10% in a period of 3–12 months was associated with a greater risk of graft failure (2).

The main cause of late allograft loss is the development of interstitial fibrosis and tubular atrophy (IF/TA) (3). These histological changes describe the final stages of different processes (CNI- toxicity, etc.) and can be detected through biopsy (4).

The “iBox,” a prediction score by the group of Loupy et al., aims to ensure a better graft monitoring and thus enable a patient-tailored diagnostic and therapy after transplantation (5). Trailin et al. were able to show that high levels of interleukin 2 in urine are associated with worsened eGFR (6). Kielar et al. showed that elevated neutrophil gelatinase-associated lipocalin (NGAL) levels in the urine were associated with an eGFR loss after transplantation (7). However, sufficient biomarkers to obtain robust and validated information about long-term allograft function and to identify patients at risk, are still lacking today.

Dickkopf 3 (DKK3) has been identified as a biomarker of kidney function in animal and clinical studies. These studies have been based on patients with acute kidney injury (AKI) or chronic kidney disease (CKD). In the context of AKI, DKK3 is currently seen as a prediction score for the development of a kidney failure (8).

DKK3, a profibrotic glycoprotein, belonging to the Dickkopf family consists of five proteins (DKK1-4, DKK like protein 1) that influence the Wnt signaling pathway through inhibition or activation. The Wnt signal pathway is an important signal transduction pathway in embryogenesis (9, 10). It is also relevant in tumor diseases (e.g., familial adenomatous polyposis) (11). In the context of kidney diseases, multiple functions are assigned, e.g., it is potentially associated with the development of ADPKD (10). DKK3 activates the canonical Wnt/b-catenin signaling pathway which induces gene expression (12).

Frederico et al. found that Dickkopf plays a role in embryonic development and was found in mesenchymal progenitor cells and mesenchymal cells. It is normally not detectable in adult cells (13). After kidney damage, DKK3 is expressed in the tubular epithelial cells and causes a profibrotic T-cell response (13). It can therefore be detected in the urine.

Studies on patients with CKD have shown that high Dickkopf values are associated with the increased incidence of tubulointerstitial fibrosis. Frederico et al. were able to show that DKK3 deficient mice showed less pronounced tubular atrophy and an improved kidney. This effect could also be demonstrated by an antibody-mediated blockade of DKK3 (13). Another study showed that higher Dickkopf values were associated with impaired kidney function and patients with high DKK3 values showed more tubulointerstitial fibrosis. The authors conclude that Dickkopf can be used as a biomarker for patients with a rapid eGFR loss over time, regardless of the underlying kidney disease (14).

The role of Dickkopf 3 in the context of kidney transplantation is completely unclear. The aim of our study was to analyze DKK3 in the urine of transplanted patients, which represents the first investigation realized in such a cohort. For this purpose, a highly standardized cohort of kidney transplant recipients was investigated. In addition to kidney function, represented by creatinine, eGFR and albuminuria, both donor and recipient specific influencing factors (e.g., age, PRA level) were analyzed. The goal of our study was to determine if a correlation between the DKK3 values and graft function over the observation period of 3 years exists and if DKK3 could be further developed as a non-invasive marker to identify patients at high risk for a deterioration in transplant function.

## Materials and Methods

### Patients' Baseline Characteristics

All patients being transplanted at our center between January 1, 2016 and December 31, 2018 were included (*n* = 122). All recipient-related data and transplantation-associated parameter were collected and archived as part of the “Regensburger Transplantationsnachsorge.” This retrospective study was approved by the Ethical Committee of the University of Regensburg.

Baseline data of the recipients were recorded and the graft function represented by creatinine, eGFR (CKD-EPI) and urinary albumin/creatinine ratio up to 36 months after transplantation was analyzed. Each recipient was grouped according to its underlying immunological risk profile (CDC-PRA, DSA, etc.) before transplantation and thereafter treated by a pre-defined immunological algorithm (15). Induction therapy was done with a CD25 monoclonal antibody basiliximab (Novartis) in patients with low and medium risk and rATG (Sanofi) in high risk patients. Maintenance immunosuppression consisted of a calcineurin inhibitor (tacrolimus), a proliferation inhibitor (mycophenolic acid) and steroids (15). DKK3 was measured non-invasively in the urine 14 days, 3, 12, 18, 24, 30, and 36 months postTx. Since the majority of the patients were anuric at the time of transplantation, the determination of DKK3 on day 0 was dispensed.

### DKK3 ELISA Analysis

Urinary midstream samples were collected from patients at the mentioned time points. The urine samples were stored at −80°C. DKK3 was measured with a commercially available ELISA according to the manufacturers' recommendations (DiaRen, Homburg, Germany). Urine samples were centrifuged at 370 g for 10 min. 100 μL of supernatant was mixed with 900 μL of sample buffer and 100 μL of the dilution was transferred to a microtiter plate coated with capture antibody and incubate for 30 min (23 ± 3°C). After repetitive washing (3x), the detection antibody was loaded with streptavidin-horseradish peroxidase (HRP) conjugate and rinsed again (3x). Substrate solution (100 μL of TMB/tetramethylbenzidine) was added and incubated for 30 min at room temperature. Finally, 100 μL of stop solution per well was added and the plate was immediately measured at 450 nm. For each microtiter plate, 6 standards were carried in duplicate at DKK3 concentrations of 0, 30, 85, 245, 700, and 2,000 pg/mL. Concentration data in urine are not very meaningful because the results depend on the dilution state of the urine. Accordingly, urinary DKK3 levels were normalized to urinary creatinine concentrations to account for dilution of the urine (14). To exclude any bias, DKK3 in all samples was measured in a blinded manner.

### Statistical Analysis

Descriptive analyses were done using absolute and percentual frequency (n, %), mean ± standard deviation, and median with corresponding interquartile range (IQR).

The course of DKK3, creatinine, eGFR, and albuminuria values from 14 days up to 3 years after transplantation were presented.

Mann- Whitney- *U*-tests were used to compare DKK3 crea ratio 3 months and 12 months postTx between patients treated with basiliximab or rATG. The time point 14d was excluded due to the presumably influence of reperfusion ischemia damage.

The associations between DKK3 crea ratio and graft function were assessed by three separate mixed linear models (MLM) including the measurement time points 12, 24, 30, and 36 months. It was examined whether the 3 or 12 month DKK3 value can predict graft function in the following course using six separate MLMs. The influence of changes in DKK3 values from month 3 to month 12 after transplantation on subsequent graft function 24, 30, and 36 month after transplantation was assessed by three separate MLMs. Changes in DKK3 were dichotomized in worse (≥ 25% increase) and good (<25% increase). With these analyses, we investigated whether DKK3 represents an independent influencing factor on kidney function and if a change in DKK3 kinetics is relevant. 25% was chosen as cut-off, in accordance to the classification of an AKI, where a 25% deterioration in kidney function is classified as stage 1 (RIFLE criteria) (16). In the context of transplantation, a deterioration in creatinine of 0.3 mg/dl is considered relevant. This also corresponds to a loss of ~25%.

The MLMs included factors that that are well-known to affect graft function, namely highest PRA level (17), cold and warm ischemia time (18, 19) and donor characteristics as age, hypertension, diabetes, and last creatinine (20, 21). MLM replaces missing values by using maximum likelihood estimates. All patients, even with missing graft function values at specific time points could be used for the analysis. Unstructured covariance type was used. As creatinine and albuminuria values were not normally distributed, values were logarithmised.

Statistical analyses were conducted with SPSS Statistics 26 (SPSS Inc, Chicago, Illinois). The level of significance was set at p two-sided ≤ 0.050. No adjustments for multiple testing were done.

## Results

### Patients' Baseline Characteristics

A total of 122 patients were transplanted with 85 being men (70%) and 37 (30%) being women. Induction therapy with basiliximab was carried out in 82 patients (67%) and 39 patients received rATG (32%). One patient received no induction therapy (1%). The mean donor age was 56 years (IQR, 47–62). The cold ischemia time averaged 480 min (IQR, 166.3–679.3), the warm ischemia time was 42 min (IQR, 33–52). Donor-specific antibodies were detected in 11 patients prior to transplantation (9%). Out of the 122 transplants performed, 41 were from a living donation (33.6%) from which 17 were from blood relatives (41.5%) and 81 were from a cadaveric donation (66.4%). Forty-nine patients received an organ from a donor with extended donor criteria (ECD) (40.2%). Eight patients died during the follow-up. Further information are shown in Table 1.

**Table 1:** Baseline characteristics of the study cohort.

	**Study cohort (*n* = 122)**
Donor- age (years)	53 ± 16
Donor- weight (kg)	78 ± 18.4
Donor- height (cm)	171.1 ± 13.2
Donor- sex (M:F)	57:65
Donor—hypertension (*n*/%)	42 (34.4)
Donor—diabetes (*n*/%)	10 (8.2)
Donor—last creatinine (median, IQR)	0.81 (0.68–1.08)
Recipient- weight (kg)	78.6 ± 14.1
Recipient- height (cm)	171.7 ± 9.2
Recipient- sex (M:F)	85:37
Re-Tx (*n*)	8 (7%)
**Cause of end stage renal disease**
ADPKD	20 (16%)
IgA- Nephropathy	24 (20%)
Hypertensive nephropathy	23 (19%)
Diabetic nephropathy	11 (9%)
Other	44 (36%)
**Mismatch**
HLA-A	0.81 ± 0.74
HLA-B	1.08 ± 0.75
HLA-DR	1.01 ± 0.7
PRA (%)- current	5.6 ± 21.2
PRA (%)- highest	13.5 ± 28.1
**Ischemia time**
Cold ischemia time (min)	475.6 ± 297.6
Warm ischemia time (min)	44.6 ± 16.7
**Rejection (** ***n*** **)**
TCMR	13 (11%)
AMR	5 (4%)
Borderline	4 (3%)
***De-novo*** **Donor specific antibodies**
HLA class I (*n*/%)	6 (5%)
HLA class II (*n*/%)	10 (8%)
Graft loss (*n*/%)	6 (5%)
Death (*n*/%)	8 (7%)

### Urinary DKK3 Crea Ratio and Resulting Allograft Function

#### DKK3 Crea Ratio

**Figure 1:**
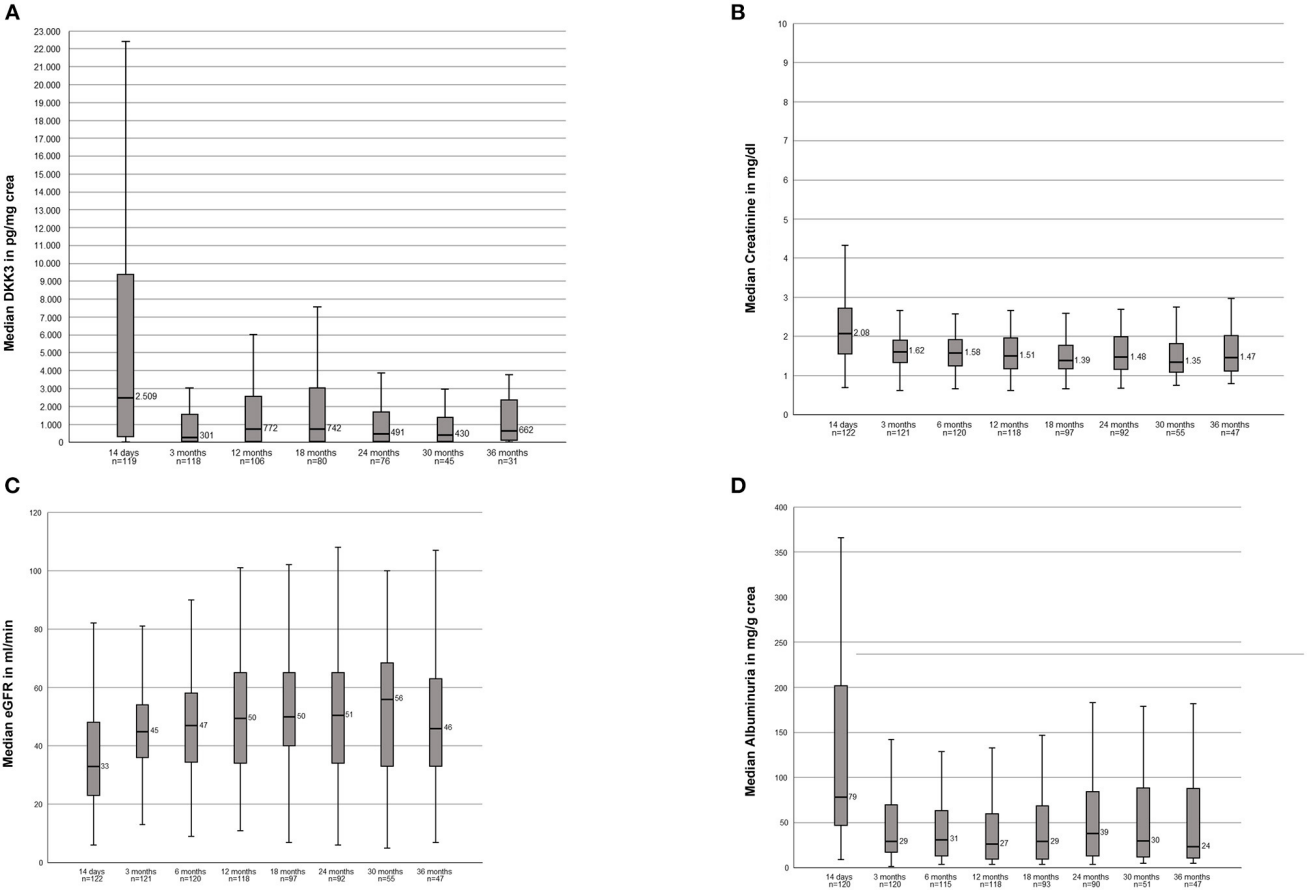
**(A)** Course of the DKK3 values over the observation period of 3 years of the entire study population. **(B)** Course of the serum creatinine values over the observation period of 3 years of the entire study population. **(C)** Course of the eGFR over the observation period of 3 year of the entire study populations. **(D)** Course of the albuminuria over the observation period of 3 years of the entire study population.

We analyzed the course of DKK3 over the observation period of all transplanted patients. The highest DKK3 value with a median of 2,509 pg/mg crea (IQR, 321-9636) were measured after 14 days. In the further course the following median values were measured: 3 months: 300.5 pg/mg crea (IQR, 33–1567); 12 months: 771.5 pg/mg crea (IQR, 45–2589); 18 months 742 pg/mg crea (IQR, 43–3059); 24 months: 491 pg/mg crea (IQR, 43–1693); 30 months: 430 pg/mg crea (IQR, 41–1521); and 36 months: 661 pg/mg crea (IQR, 83–2526) (Figure 1A).

#### Kidney Function Values of the Entire Study Population

Stable creatinine values over the entire 36 months were seen: 14d: 2.08 mg/dl (1.54–2.73); 3 months: 1.62 mg/dl; (1.34–1.92) 12 months: 1.51 mg/dl (1.18–1.96); 18 months: 1.39 mg/dl (1.17–1.82); 24 months: 1.48 mg/dl (1.16–2.01); 30 months: 1.35 mg/dl (1.05–1.84); 36 months: 1.47 mg/dl (1.11–2.03) (Figure 1B). In accordance resulting eGFR values were also stable within the observation period: 14d: 33 ml/min (22.8–48); 3 months: 45 ml/min (36–54.5); 12 months: 49.5 (34–63.5), 18 months: 50 ml/min (39.5–65); 24 months: 50.5 (34–65); 30 months: 56 ml/min (33–70); 36 months: 46 ml/min (32–64) (Figure 1C).

The following albuminuria values were measured: 14d: 78.50 mg/g crea (46.9–201.75); 3 months: 29.35 mg/g crea (16.95–70.40); 12 months: 26.75 mg/g crea (9.42–60.93); 18 months: 29.30 mg/g crea (9.63–69.85); 24 months: 38.50 mg/g crea (13–85.08); 30 months: 29.90 mg/g crea (11.2–90.60); 36 months: 23.80 mg/g crea (10.3–89.50). Two patients showed clear outliers at the time points 24 months (10,365 mg/g crea) and 30 months (11,079 mg/g crea). In both patients the albuminuria was associated with a subsequent graft loss (Figure 1D).

### Impact of Chosen Induction Therapy on DKK3

**Figure 2:**
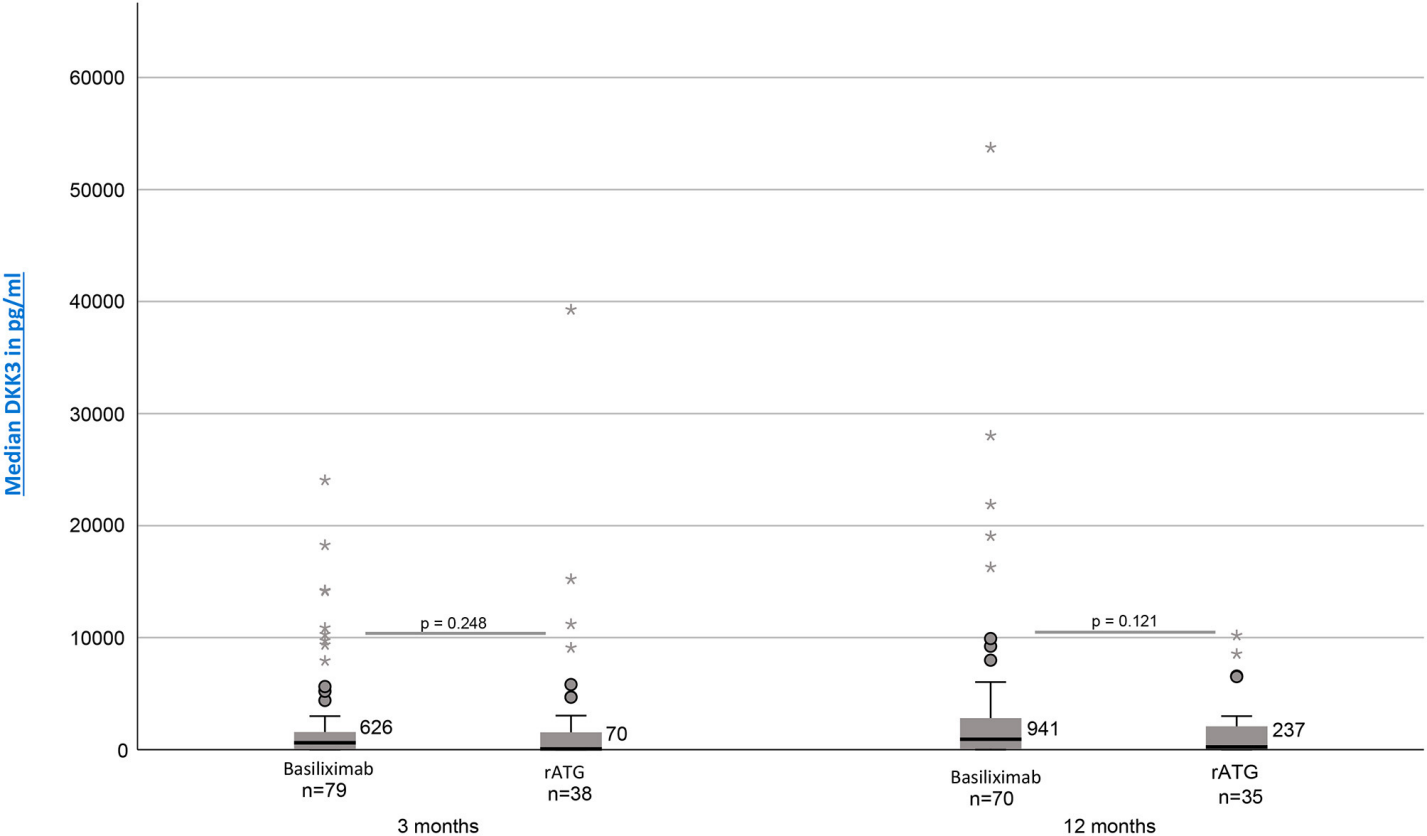
DKK3 values at time point 3 and 12 months depending on induction therapy (Basiliximab vs. rATG).

Investigation whether the chosen induction therapy had an impact on the resulting DKK3 values showed no statistically significant differences. However, patients treated with basiliximab showed 3 and 12 month postTx continuously higher DKK3 values [3 months: median = 626 pg/mg crea (IQR, 38–1580, *n* = 79); 12 months: median = 941 pg/mg crea (IQR, 75–2837, *n* = 70)] than patients treated with rATG [3 months: median =70 pg/mg crea (IQR, 18–1761, *n* = 38); 12 months: median = 237 pg/mg crea (IQR, 32–2155, *n* = 35)] (3 months: *p* = 0.248; 12 months: *p* = 0.121) (Figure 2).

### Impact of DKK3 on Simultaneously Measured Allograft Function

The mixed linear models showed that higher donor age (*p* < 0.001) and DKK3 expression level (*p* = 0.011) were significantly associated with impaired graft function. More precisely, if the donor age increased by 10 years, resulting creatinine increased by 0.11 mg/dl and if the DKK3 increased by 10.000, creatinine increased by 0.16 (95% CI 0.09–0.23). The analysis of the eGFR showed that donor age (*p* < 0.001) and last donor creatinine (*p* = 0.03) were the only influencing factor, whereas DKK3 did not reach the level of significance (*p* = 0.13). In the case of albuminuria, both donor age (*p* < 0.001), donor diabetes (*p* = 0.03) and the DKK3 value (*p* < 0.001) were statistically significant (Tables 1a–c of the Supplement).

### Prediction of Subsequent Graft Function by DKK3

It was examined whether 3 or 12 month DKK3 values could predict subsequent allograft function. Higher DKK3 values 3 and 12 months after transplantation predicted higher subsequent creatinine values (*p* < 0.050) up to 36 months. Moreover, higher DKK3 values 3 and 12 months postTx predicted lower subsequent eGFR values (*p* < 0.050) in the same observation period. Higher DKK3 values 3 months after transplantation predicted higher albuminuria values 6 months (*p* = 0.013) and 12 months postTx (*p* = 0.050), but not on a later time point (*p* > 0.050). Higher DKK3 values 12 months after transplantation predicted higher subsequent albuminuria values up to month 36 (*p* < 0.050). Donor age was the only consistently significant parameter associated with graft function (*p* < 0.050), whereas the other analyzed parameters showed no consistent influence. More precisely, an increase in donor age by 10 years lead to a creatinine increase of 0.13 mg/dl, while an increase in DKK3 by 10.000 lead to a creatinine increase of 0.58 mg/dl (Tables 2a–f of the Supplement).

### Impact of DKK3 Kinetics for Allograft Function

Comparing patients with a DKK3 increase ≥ 25% from time 3 to 12 months and patients with a decrease or an increase of <25% in the same period, patients with an increase of ≥ 25% in DKK3 values showed higher creatinine values (*p* = 0.038), a lower eGFR (*p* = 0.018) and higher albuminuria values (*p* = 0.005) for subsequent time points. These associations could be confirmed for graft function 30, and 36 months postTx (*p* < 0.050), except for albuminuria values 36 months after transplantation (*p* = 0.092) (Tables 2A–C). Roughly shown, less intense DKK3 increase between 3 and 12 months resulted in an eGFR differences of about 9–10 ml/min and in a 7–12 times lower albuminuria over the observation period (24 till 36 months) in contrast to patients with a DKK3 increase ≥ 25%.

**Table 2:** **(A)** Medium creatinine values depending on the DKK3 change of month 3–12 (≥ 25 vs. <25%).

**Time DKK3 change**		**Mean value**	**Confidence interval 95%**	
			**Upper limit**	**Lower limit**
**(A)**				
24 months	<25%	1.56	1.22	1.89
	≥ 25%	1.98	1.67	2.29
30 months	<25%	1.44	1.00	1.88
	≥ 25%	2.11	1.70	2.52
36 months	<25%	1.42	1.02	1.82
	≥ 25%	2.03	1.66	2.40
**(B)**				
24 months	<25%	54.51	48.86	60.16
	≥ 25%	47.49	42.25	52.74
30 months	<25%	55.13	49.38	60.88
	≥ 25%	47.01	41.74	52.27
36 months	<25%	57.13	51.16	63.10
	≥ 25%	48.22	42.70	53.75
**(C)**				
24 months	<25%	64.25	−308.44	436.94
	≥ 25%	393.57	47.44	739.71
30 months	<25%	46.35	−353.97	446.66
	≥ 25%	403.34	31.74	774.94
36 months	<25%	44.45	−92.21	181.11
	≥ 25%	213.80	86.92	340.68

***(B)** Medium eGFR depending on the DKK3 change of month 3–12 (≥ 25 vs. <25%). **(C)** Medium albuminuria depending on the DKK3 change of month 3–12 (≥ 25 vs. <25%)*.

## Discussion

In our study, we examined the influence of Dickkopf 3 on graft function in kidney transplant recipients. We were able to show that DKK3 correlates with resulting graft function, represented by creatinine, eGFR and albuminuria, over an observation period of 36 months. DKK3 can even predict kidney function as illustrated by the association of 3 and 12 months DKK3 values and subsequent allograft function. Furthermore, changes in DKK3 values from month 3 to 12 (≥25%) were associated with a significantly deteriorated graft function, being illustrated by tremendous differences in creatinine, eGFR, and albuminuria values. Our study is the first investigation of DKK3 referring to transplantation medicine.

Regarding the function of DKK3, studies have shown that DKK3 is secreted only by stressed tubular epithelial cells in the adult kidney. Using two animal models, an adenine-induced nephropathy and a model of an unilateral ureter obstruction, Gröne et al. showed by usage of a DKK3 knockout that DKK3 deficiency leads to a marked reduction in tubular damage and renal fibrosis. DKK3 deficiency triggered an antifibrotic T cell response and reduced activity of the WNT–β-catenin signaling pathway. These results could also be reproduced by an antibody-mediated blockade of DKK3. DKK3 therefore appears to be an important mediator of renal fibrosis and thus of deterioration in renal function (9).

Schunk et al. were able to show that patients after cardiac surgery and increased DKK3 scores (>471 pg/ml) had an increased risk for developing AKI (22). A comparable observation could be reproduced in our analysis. However, our data are more closely linked with chronic changes. Patients with a DKK3 dynamic of more than 25% showed a deteriorated graft function and also more albuminuria than patients with a smaller change in DKK3. Especially the changes in albuminuria, being 7–12 times higher in patients with intensified DKK3 increase, do not only link DKK3 expression levels with allograft function, but also with arising structural damage.

Zewinger et al. were able to show that high levels of DKK3 are associated with impaired function in patients with CKD. DKK3 could be seen as a predictor for an eGFR loss independent of the underlying disease. This study showed that high DKK3 values can function as a prognostic parameter regardless of the accompanying albuminuria (14). Similar results were showen by Sanchez-Alamo. By determining DKK3 in the urine, patients with a high risk of deterioration in kidney function could be identified, regardless of the underlying disease (23). A correlation between DKK3 and creatinine as well as the eGFR was also found in our work. In contrast to Zewinger, however, a significant influence of DKK3 could also be found for albuminuria.

We were able to see a trend toward lower DKK3 values after T-cell depleting induction therapy in comparison to an immunomodulatory therapy with basiliximab. Regarding the impact of immunosuppressives on the development of DKK3, no further data are available. But in literature, the influence of DKK3 on T- lymphocytes is discussed. As already mentioned, DKK3 seems to trigger a profibrotic T cell response. Federico et al. were also able to show that after an antibody-mediated blockade of DKK3, an increased presence of protective T cells (IFNγ-producing Th1 and Tregs) can be demonstrated (13). Taking this into account, the evidence of lower DKK3 values under a T-cell depleting therapy seems understandable. Further investigations on the influence of immunosuppressives would be useful to further evaluate specific “anti-DKK3 and therefore presumable anti-fibrotic immunosuppressive protocols.”

In our cohort, we were able to recognize a total of 18 rejections over the entire observation period, both T cell-mediated and antibody-mediated rejections. Most of them occurred within the first 14 days. The analysis of the DKK3 values between patients with a rejection compared to patients without a rejection showed no statistically significant difference. However, it should be noted here that there is a relevant difference in the number of cases in the two groups as a possible confounding factor.

Our study is the first analyzing Dickkopf 3 after kidney transplantation. Nevertheless, it is a monocentric study with a limited case number. There are currently no special biomarkers postTx available to estimate the individual risk for a deterioration in graft function. DKK3 can be easily integrated into everyday clinical practice thanks to its detection in urine. Similar to Zewinger in his study, we were also able to see a clear influence of DKK3 on graft function (14). The use of DKK3 as a predictor of graft function should therefore be considered and proofed in a multi-center clinical trial. Animal studies showed that an anti-DKK3 antibody could inhibit the development of fibrosis in mice. DKK3 thus also represents a possible therapeutic target. It should be noted critically that defined cut-off values for DKK3, from which a clinical consequence must result, are still missing. Looking at our study, the determination of DKK3 at time points 3 and 12 months after transplantation could be a helpful new screening parameter in the follow-up. Nevertheless, long-term analyzes and prospective multicenter studies would be necessary in order to address the still open questions and to deepen our findings made in a single-center study.

## Significant Statement

Whereas, in the context of chronic kidney disease, Dickkopf 3 has been recognized as a marker to identify patients at risk for a progressive loss of kidney function, to date, the impact of DKK3 after transplantation has not yet been analyzed.

In our study on kidney transplant recipients, DKK3 not only could precisely predict subsequent allograft function but an increase in DKK3 values within the first year after transplantation was associated with a deterioration in allograft function.

With the presented data, DKK3 can be considered as a new indicator of impaired graft function after transplantation. However, further prospective and interventional studies are needed to verify our findings.

## Data Availability Statement

The original contributions presented in the study are included in the article/Supplementary Material, further inquiries can be directed to the corresponding author/s.

## Ethics Statement

The studies involving human participants were reviewed and approved by Ethical Committee of the University of Regensburg. The patients/participants provided their written informed consent to participate in this study.

## Author Contributions

AS, BB, and TB: concept/design. AS, PF, and TB: data collection. AS, KM, FZ, and TB: statistics. AS, LS, and TB: data analysis/interpretation. AS, LS, BB, and TB: drafting article. AS, LS, KM, FZ, PF, BB, and TB: critical revision of article and approval of article. All authors contributed to the article and approved the submitted version.

## Funding

This work was funded by the Deutsche Forschungsgemeinschaft (DFG, German Research Foundation), project number 387509280, SFB 1350 Project B6 to TB and BB.

## Conflict of Interest

The authors declare that the research was conducted in the absence of any commercial or financial relationships that could be construed as a potential conflict of interest.

## Publisher's Note

All claims expressed in this article are solely those of the authors and do not necessarily represent those of their affiliated organizations, or those of the publisher, the editors and the reviewers. Any product that may be evaluated in this article, or claim that may be made by its manufacturer, is not guaranteed or endorsed by the publisher.

## Abbreviations

DKK3: Dickkopf 3
IF/TA: Interstitial fibrosis and tubular atrophy
eGFR: Estimated glomerular filtration rate
NGAL: Neutrophil gelatinase-associated lipocalin
AKI: Acute kidney injury
CKD: Chronic kidney disease
ADPKD: Autosomal dominant polycystic kidney disease
CDC- PRA: Complement dependent cytotoxicity- Panel reactive antibody
DSA, Donor specific antibody;rATG: Rabbit anti-thymocyte globulin
HRP: Horseradish peroxidase
TMB: Tetramethylbenzidine
IQR: Interquartile range
MLM: Mixed linear models
ECD: Extended donor criteria
Tregs: Regulatory T cells.
