# Supplementary material for: Dickkopf 3—A New Indicator for the Deterioration of Allograft Function After Kidney Transplantation
Source: Front Med (Lausanne). 2022 May 11;9:885018. doi: 10.3389/fmed.2022.885018 (PMC9130628; doi:10.3389/fmed.2022.885018)
Supplement: Supplementary file 1 [file Data_Sheet_1.PDF]

**Supplement**

| Parameter | Estimate | Sig. | Confidence interval 95% | |
|---|---|---|---|---|
| | | | Lower limit | Upper limit |
| DKK3 | 1.000004 | 0.011 | 1.000001 | 1.000008 |
| Cold ischemia time | 1.000122 | 0.169 | 0.999947 | 1.000297 |
| Warm ischemia time | 1.001094 | 0.479 | 0.998042 | 1.004155 |
| Donor Age | 1.007904 | <0.001 | 1.004476 | 1.011343 |
| Donor hypertension | 1.044137 | 0.455 | 0.931479 | 1.170421 |
| Donor diabetes | 1.019655 | 0.828 | 0.854268 | 1.217062 |
| Donor last creatinine | 1.047688 | 0.144 | 0.984018 | 1.115477 |
| Highest PRA level | 1.000165 | 0.860 | 0.998319 | 1.002014 |

Table 1a. Multivariate analysis regarding the influence of DKK3, cold and warm ischemia time as well as donor age and PRA level on the simultaneously measured creatinine values.

| Parameter | Estimate | Sig. | Confidence interval 95% | |
|---|---|---|---|---|
| | | | Lower limit | Upper limit |
| DKK3 | -0.000152 | 0.125 | -0.000348 | 0.000043 |
| Cold ischemia time | -0.006476 | 0.142 | -0.015145 | 0.002193 |
| Warm ischemia time | -0.105568 | 0.165 | -0.255061 | 0.043925 |
| Donor Age | -0.587250 | <0.001 | -0.756019 | -0.418480 |
| Donor hypertension | -2.029244 | 0.478 | -7.679181 | 3.620692 |
| Donor diabetes | -5.008815 | 0.261 | -13.794421 | 3.776791 |
| Donor last creatinine | -3.396888 | 0.031 | -6.482900 | -0.310875 |
| Highest PRA level | -0.039762 | 0.380 | -0.129207 | 0.049682 |

Table 1b. Multivariate analysis regarding the influence of DKK3, cold and warm ischemia time as well as donor age and PRA level on the simultaneously measured eGFR.

| | | | Confidence interval 95% | |
|---|---|---|---|---|
| Parameter | Estimate | Sig. | Lower limit | Upper limit |
| DKK3 | 1.000041 | <0.001 | 1.000023 | 1.000060 |
| Cold ischemia time | 0.999543 | 0.162 | 0.998900 | 1.000187 |
| Warm ischemia time | 1.006901 | 0.228 | 0.995655 | 1.018275 |
| Donor Age | 1.027440 | <0.001 | 1.014598 | 1.040444 |
| Donor hypertension | 0.735599 | 0.148 | 0.484294 | 1.117310 |
| Donor diabetes | 2.047269 | 0.032 | 1.065001 | 3.935501 |
| Donor last creatinine | 1.185221 | 0.140 | 0.945208 | 1.486179 |
| Highest PRA level | 1.003441 | 0.318 | 0.996662 | 1.010267 |

Table 1c. Multivariate analysis regarding the influence of DKK3, cold and warm ischemia time as well as donor age and PRA level on the simultaneously measured albuminuria.

| Parameter | Estimate | Sig. | Confidence interval 95% | |
|---|---|---|---|---|
| | | | Lower limit | Upper limit |
| 6Mo*DKK3 3Mo | 1.000024 | <0.001 | 1.000014 | 1.000035 |
| 12Mo*DKK3 3Mo | 1.000019 | <0.001 | 1.000009 | 1.000030 |
| 24Mo*DKK3 3Mo | 1.000022 | 0.001 | 1.000010 | 1.000035 |
| 30Mo*DKK3 3Mo | 1.000019 | 0.015 | 1.000004 | 1.000035 |
| 36Mo*DKK3 3Mo | 1.000022 | 0.005 | 1.000007 | 1.000037 |
| Cold ischemia time | 1.000187 | 0.053 | 0.999998 | 1.000376 |
| Warm ischemia time | 1.001204 | 0.470 | 0.997918 | 1.004500 |
| Donor Age | 1.008808 | <0.001 | 1.005099 | 1.012531 |
| Donor hypertension | 1.055032 | 0.393 | 0.932275 | 1.193953 |
| Donor diabetes | 1.073478 | 0.461 | 0.887841 | 1.297930 |
| Donor last creatinine | 1.056013 | 0.106 | 0.988312 | 1.128352 |
| Highest PRA level | 1.000661 | 0.502 | 0.998719 | 1.002607 |

Table 2a. Multivariate analysis regarding the influence of DKK3, cold and warm ischemia time as well as PRA level and donor age at the time point 3 months on the subsequent creatinine values (6, 12, 24, 30, 36 months).

| Parameter | Estimate | Sig. | Confidence interval 95% | |
|---|---|---|---|---|
| | | | Lower limit | Upper limit |
| 6Mo*DKK3 3Mo | -0.000978 | <0.001 | -0.001484 | -0.000473 |
| 12Mo*DKK3 3Mo | -0.000817 | 0.002 | -0.001324 | -0.000311 |
| 24Mo*DKK3 3Mo | -0.000820 | 0.008 | -0.001420 | -0.000220 |
| 30Mo*DKK3 3Mo | -0.000787 | 0.030 | -0.001498 | -0.000076 |
| 36Mo*DKK3 3Mo | -0.000850 | 0.016 | -0.001538 | -0.000161 |
| Cold ischemia time | -0.009202 | 0.058 | -0.018703 | 0.000298 |
| Warm ischemia time | -0.094664 | 0.257 | -0.259436 | 0.070107 |

| | | | | |
|---|---|---|---|---|
| Donor Age | -0.629673 | <0.001 | -0.814525 | -0.444820 |
| Donor hypertension | -4.326188 | 0.171 | -10.543028 | 1.890652 |
| Donor diabetes | -5.976571 | 0.217 | -15.513785 | 3.560643 |
| Donor last creatinine | -2.734684 | 0.108 | -6.077138 | 0.607770 |
| Highest PRA level | -0.040289 | 0.414 | -0.137616 | 0.057037 |

Table 2b. Multivariate analysis regarding the influence of DKK3, cold and warm ischemia time as well as PRA level and donor age at the time point 3 months on the subsequent eGFR (6, 12, 24, 30, 36 months).

| | | | Confidence interval 95% | |
|---|---|---|---|---|
| Parameter | Estimate | Sig. | Lower limit | Upper limit |
| 6Mo*DKK3 3Mo | 1.000047 | 0.013 | 1.000010 | 1.000084 |
| 12Mo*DKK3 3Mo | 1.000041 | 0.050 | 1.000000 | 1.000082 |
| 24Mo*DKK3 3Mo | 1.000030 | 0.215 | 0.999982 | 1.000078 |
| 30Mo*DKK3 3Mo | 1.000011 | 0.739 | 0.999947 | 1.000074 |
| 36Mo*DKK3 3Mo | 1.000025 | 0.440 | 0.999960 | 1.000091 |
| Cold ischemia time | 0.999737 | 0.440 | 0.999063 | 1.000410 |
| Warm ischemia time | 1.004525 | 0.455 | 0.992605 | 1.016587 |
| Donor Age | 1.030490 | <0.001 | 1.017015 | 1.044143 |
| Donor hypertension | 0.764686 | 0.233 | 0.490947 | 1.191055 |
| Donor diabetes | 2.388871 | 0.014 | 1.195066 | 4.775221 |
| Donor last creatinine | 1.089807 | 0.473 | 0.860091 | 1.380877 |
| Highest PRA level | 1.002443 | 0.491 | 0.995459 | 1.009475 |

Table 2c. Multivariate analysis regarding the influence of DKK3, cold and warm ischemia time as well as PRA level and donor age at the time point 3 months on the subsequent albuminuria (6, 12, 24, 30, 36 months).

| Parameter | Estimate | Sig. | Confidence interval 95% | |
|---|---|---|---|---|
| | | | Lower limit | Upper limit |
| 24Mo*DKK3 12Mo | 1.000029 | <0.001 | 1.000018 | 1.000039 |
| 30Mo*DKK3 12Mo | 1.000032 | <0.001 | 1.000021 | 1.000043 |
| 36Mo*DKK3 12Mo | 1.000030 | <0.001 | 1.000019 | 1.000041 |
| Cold ischemia time | 1.000031 | 0.816 | 0.999769 | 1.000293 |
| Warm ischemia time | 1.003987 | 0.098 | 0.999244 | 1.008752 |
| Donor Age | 1.008923 | 0.003 | 1.003215 | 1.014663 |
| Donor hypertension | 1.182390 | 0.049 | 1.000896 | 1.396794 |
| Donor diabetes | 1.057167 | 0.673 | 0.814285 | 1.372497 |
| Donor last creatinine | 1.063907 | 0.165 | 0.974429 | 1.161601 |
| Highest PRA level | 1.002397 | 0.157 | 0.999059 | 1.005746 |

Table 2d. Multivariate analysis regarding the influence of DKK3, cold and warm ischemia time as well as PRA level and donor age at the time point 12 months on the subsequent creatinine values (24, 30, 36 months).

| Parameter | Estimate | Sig. | Confidence interval 95% | |
|---|---|---|---|---|
| | | | Lower limit | Upper limit |
| 24Mo*DKK3 12Mo | -0.000880 | 0.001 | -0.001378 | -0.000382 |
| 30Mo*DKK3 12Mo | -0.000957 | <0.001 | -0.001442 | -0.000472 |
| 36Mo*DKK3 12Mo | -0.000924 | <0.001 | -0.001415 | -0.000432 |
| Cold ischemia time | -0.004125 | 0.500 | -0.016223 | 0.007973 |
| Warm ischemia time | -0.108914 | 0.333 | -0.331168 | 0.113340 |
| Donor Age | -0.713185 | <0.001 | -0.969977 | -0.456394 |
| Donor hypertension | -8.772740 | 0.025 | -16.396416 | -1.149064 |
| Donor diabetes | -2.325552 | 0.701 | -14.310823 | 9.659719 |
| Donor last creatinine | -4.538319 | 0.027 | -8.541245 | -0.535392 |
| Highest PRA level | -0.133086 | 0.095 | -0.289796 | 0.023625 |

Table 2e. Multivariate analysis regarding the influence of DKK3, cold and warm ischemia time as well as PRA level and donor age at the time point 12 months on the subsequent eGFR (24, 30, 36 months).

| Parameter | Estimate | Sig. | Confidence interval 95% | |
|---|---|---|---|---|
| | | | Lower limit | Upper limit |
| 24Mo*DKK3 12Mo | 1.000047 | 0.016 | 1.000009 | 1.000086 |
| 30Mo*DKK3 12Mo | 1.000047 | 0.028 | 1.000005 | 1.000088 |
| 36Mo*DKK3 12Mo | 1.000048 | 0.024 | 1.000007 | 1.000090 |
| Cold ischemia time | 1.000292 | 0.540 | 0.999348 | 1.001237 |
| Warm ischemia time | 0.998527 | 0.868 | 0.981158 | 1.016204 |
| Donor Age | 1.038768 | <0.001 | 1.018526 | 1.059413 |
| Donor hypertension | 0.960825 | 0.893 | 0.531164 | 1.738040 |
| Donor diabetes | 2.546557 | 0.045 | 1.021803 | 6.346580 |
| Donor last creatinine | 1.311023 | 0.085 | 0.962125 | 1.786443 |
| Highest PRA level | 1.000837 | 0.898 | 0.987966 | 1.013875 |

Table 2f. Multivariate analysis regarding the influence of DKK3, cold and warm ischemia time as well as PRA level and donor age at the time point 12 months on the subsequent albuminuria (24, 30, 36 months).
